# Higher Lipopolysaccharide Levels Are Linked to Less Anhedonia and Lower Severity of Major Depressive Disorder

**DOI:** 10.3390/brainsci16080852

**Published:** 2026-08-12

**Authors:** Egle Milasauskiene, Julius Burkauskas, Simonas Jesmanas, Rymante Gleizniene, Vilmante Borutaite, Nijole Raskauskiene, Kristina Skemiene, Virginija Adomaitiene, Brigita Gradauskiene, Guy C. Brown, Vesta Steibliene

**Affiliations:** 1 Laboratory of Behavioral Medicine, Neuroscience Institute, Lithuanian University of Health Sciences, LT-50161 Kaunas, Lithuania; julius.burkauskas@lsmu.lt (J.B.); nijole.raskauskiene@lsmu.lt (N.R.); vesta.steibliene@lsmu.lt (V.S.); 2 Department of Psychiatry, Lithuanian University of Health Sciences, LT-50161 Kaunas, Lithuania; virginija.adomaitiene@lsmu.lt; 3 Department of Radiology, Lithuanian University of Health Sciences, LT-50161 Kaunas, Lithuania; simonas.jesmanas@lsmu.lt (S.J.); rymante.gleizniene@lsmu.lt (R.G.); 4 Laboratory of Biochemistry, Neuroscience Institute, Lithuanian University of Health Sciences, LT-50161 Kaunas, Lithuania; vilmante.borutaite@lsmu.lt (V.B.); kristina.skemiene@lsmu.lt (K.S.); 5 Department of Immunology and Allergology, Lithuanian University of Health Sciences, LT-50161 Kaunas, Lithuania; brigita.gradauskiene@lsmu.lt; 6 Department of Biochemistry, University of Cambridge, Cambridge CB21QW, UK; gcb3@cam.ac.uk

**Keywords:** major depressive disorder, immune dysregulation, lipopolysaccharides, endotoxins, anhedonia

## Abstract

**Highlights:**

**What are the main findings?**

- Higher LPS levels were associated with lower depression severity, and the associations between depressive symptoms and LPS were strongest at higher LPS levels.
- Anhedonia mediated the association between LPS and depression severity.

**What are the implications of the main findings?**

- The severity of anhedonia may help distinguish a biologically distinct subgroup of patients with LPS-associated MDD.
- The association between LPS and depressive symptoms supports investigating LPS-lowering interventions for patients with LPS-associated depression.

**Abstract:**

**Background:** Inflammatory mechanisms contribute to major depressive disorder (MDD) in a subset of patients. Lipopolysaccharide (LPS), a potent inflammatory endotoxin, can induce depressive-like symptoms, but its relationship with specific depressive symptom profiles remains unclear. **Methods:** In total, 95 adults with MDD (mean age was 44 ± 14 years) were recruited from psychiatric inpatient and outpatient services. Depressive symptom severity was assessed using the Montgomery–Åsberg Depression Rating Scale (MADRS). Serum concentrations of LPS, IL-10, TNF-α, IL-6, and IFN-γ were measured by ELISA. Associations between inflammatory markers and depressive symptoms were examined using group comparisons, correlation analyses, multivariable linear regression, quantile regression, and mediation analyses adjusted for demographic and clinical covariates. **Results:** Patients with severe depression exhibited significantly lower circulating LPS levels than those with mild-to-moderate depression (95.6 vs. 165.2 pg/mL, *p* = 0.007), despite no significant differences in cytokine concentrations. LPS levels were inversely associated with depression severity, anhedonia factor score, and MADRS item 8 (“inability to feel”). Lower LPS concentrations were independently predicted by higher inability to feel, reduced appetite and combined pharmacotherapy use. Quantile regression demonstrated heterogeneity across the LPS distribution, with symptom–LPS associations evident among patients with higher LPS concentrations. Higher LPS levels were associated with greater apparent sadness, concentration difficulties, and reduced sleep, but lower levels of anhedonia, less reduced appetite, less lassitude, lower inner tension, and lower suicidality. Mediation analyses indicated that anhedonia mediated the association between LPS levels and depression severity. **Conclusions:** Higher circulating LPS may characterize a biologically distinct subgroup of MDD patients with lower symptom severity and less anhedonia, supporting biological heterogeneity within MDD.

## 1. Introduction

There is increasing evidence that inflammation is associated with and may contribute to depressive symptoms in at least a proportion of patients with major depressive disorder (MDD) [1,2,3,4,5,6,7]. Thus, it has been proposed that there is an inflammatory subtype of depression [3,5]. One potential cause of this inflammation is lipopolysaccharide (LPS, also known as endotoxin), which can potently induce inflammation via Toll-like receptor 4 and other receptors [8,9,10,11]. LPS mainly originates from Gram-negative bacteria in the gut but can cross the gut wall in various conditions to induce inflammation in the body [12]. LPS in the blood is known as ‘endotoxemia’. Injection of LPS into the blood rapidly induces depressive symptoms in both human volunteers and animals, and thus, LPS injection is used as an experimental model of depression [13,14,15]. In animal studies, LPS administration induces depressive-like behaviors such as reduced activity and motivation [16,17]. In humans, administration of LPS at doses of 0.4–1.0 ng/kg rapidly induces a range of symptoms resembling those observed in depression, commonly referred to as “sickness behavior”; however, it does not reliably induce anhedonia [18,19,20,21,22,23,24].

Previous studies have reported elevated circulating LPS levels in several populations with depression, including adults [25], pregnant women [26], and adolescents [27]. These studies consistently found higher circulating LPS concentrations in participants with MDD than in comparison groups. However, none investigated the relationships between circulating LPS levels and specific depressive symptoms or symptom severity. Recent evidence suggests that peripheral inflammatory alterations in MDD are heterogeneous and may vary across clinical subtypes and symptom profiles [28,29]. In our previous study, we found that circulating LPS levels were elevated in adults with MDD compared with healthy controls (HCs) [30]; however, this increase was observed only in a subgroup of patients, whereas most patients had LPS levels comparable to those of HCs [30]. Furthermore, patients with more severe depression exhibited lower LPS levels than those with mild-to-moderate depression [30]. In the same study, we also demonstrated an altered inflammatory profile in MDD, characterized by increased TNF-α and decreased IL-10 concentrations, whereas IL-6 and IFN-γ levels did not differ significantly between patients with MDD and HCs [30]. These findings suggested that elevated LPS may characterize a distinct subgroup of patients with MDD and provided the rationale for the present study, which examined whether circulating LPS and cytokines are associated with specific depressive symptoms and depression severity subgroups, rather than overall depression severity.

## 2. Materials and Methods

### 2.1. Study Design and Population

This analysis is part of a cross-sectional study [31], conducted at the Lithuanian University of Health Sciences Hospital, Kaunas Clinics (Psychiatry Clinic, inpatient department, and Nervous System Diseases Outpatient Department). Adult patients consecutively admitted for treatment and diagnosed with MDD between June 2022 and December 2024 were invited to participate in the study. A total of 100 participants met the inclusion criteria and were enrolled.

Inclusion criteria were: (1) MDD diagnosis according to DSM-5, confirmed by a psychiatrist, and (2) age ≥ 18 years. Exclusion criteria were: (1) comorbid psychiatric disorders and/or substance use disorders (except tobacco) within the past year; (2) somatic conditions affecting inflammatory processes; (3) infection, vaccination, or antibiotic/anti-inflammatory medication use within the past month; and (4) pregnancy or breastfeeding.

Participants attended a single study visit and completed an investigator-designed questionnaire assessing sociodemographic characteristics, smoking status, body mass index (BMI), medical and psychiatric history, treatment, and suicidal behavior.

### 2.2. Assessment of Depressive Symptoms

Depressive symptom severity was assessed using the Structured Interview Guide for the Montgomery–Åsberg Depression Rating Scale (MADRS-SIGMA) [32,33]. The MADRS-SIGMA consists of 10 items, each rated on a Likert-type scale from 0 to 6 (0 = no symptoms, 6 = very severe symptoms). The total score ranges from 0 to 60, with higher scores indicating greater symptom severity. Participants were categorized according to established MADRS cut-offs as follows: 8–17 (mild), 18–34 (moderate), and 35–60 (severe) [34]. Following a previous study [35], MADRS item 8 (“inability to feel”) was used to evaluate anhedonia. Concordance with the Snaith–Hamilton Pleasure Scale (SHAPS), a validated measure of anhedonia [36], supports the use of item 8 as a proxy for anhedonia in depressed populations, as it shows the strongest correlation with SHAPS among all MADRS items [37]. Additionally, the anhedonia factor score (sum of MADRS items 1 (“apparent sadness”), item 2 (“reported sadness”), item 6 (“concentration difficulties”), item 7 (“lassitude”), item 8 (“inability to feel”)) [38,39] was included for comparison; however, it reflects a broader depressive symptom domain. Additionally, we evaluated changes in neurovegetative symptoms (MADRS item 4 (“reduced sleep”) and MADRS item 5 (“reduced appetite”)) as they are prominent symptoms of the sickness behavior that can be induced by LPS [40,41].

### 2.3. Blood Sample Collection and Analysis

Peripheral blood samples (16 mL) were collected in the morning after an overnight fast of at least 8 h into serum tubes containing a clot activator. The samples were kept on ice and processed no sooner than 30 min after collection. Serum was separated by centrifugation at 1500× *g* for 15 min at 4 °C, aliquoted into endotoxin-free polypropylene tubes, immediately frozen, and stored at −80 °C until analysis. All analyses were performed using first-thawed aliquots, and repeated freeze–thaw cycles were avoided. Due to hemolysis, three samples were excluded, resulting in 97 patients included in the biochemical analyses.

Serum LPS concentrations were measured using a commercially available sandwich ELISA kit (Antibodies, Limerick, PA, USA; catalogue number ABIN6975836). Absorbance was read with a Multiskan GO plate reader (Thermo Fisher Scientific, Waltham, MA, USA), and concentrations (pg/mL) were calculated from a standard curve generated using purified *E. coli* J5 LPS. The assay detection range was 6.25–400 pg/mL. All samples were above the lower limits of detection and quantification. Measurements were performed in duplicate, and mean values were used for analysis. Intra- and inter-assay coefficients of variation (CVs) were <8% and <10%, respectively.

Cytokine concentrations were measured using commercially available sandwich ELISA kits with biotin-labelled antibodies, according to the manufacturers’ instructions. TNF-α (BioVendor R&D, Brno, Czech Republic; catalogue number RAF128R) and IL-6 (BioVendor R&D, Brno, Czech Republic; catalogue number RD194015200R), as well as IL-10 (Thermo Fisher Scientific, Waltham, MA, USA; catalogue number EHIL10) and IFN-γ (Thermo Fisher Scientific, Waltham, MA, USA; catalogue number KHC4021), were analyzed in duplicate. Intra- and inter-assay CVs were below 10% for all assays.

### 2.4. Statistical Analyses

Statistical analyses were performed using SPSS 30.0 (IBM, Chicago, IL, USA) and JASP software (version 0.95.4). Two outliers were identified and excluded prior to final analyses. Outliers were identified using Tukey’s method, defined as observations lying below Q1 − 1.5 × IQR or above Q3 + 1.5 × IQR [42]. The normality of continuous variables, including plasma biomarker concentrations, was assessed using histograms and the Shapiro–Wilk test. Variables with a Shapiro–Wilk *p*-value > 0.05 were considered normally distributed. For variables that deviated from normality, non-parametric statistical methods were used as appropriate; no log transformation was applied. Non-normally distributed variables are reported as median (Q1–Q3). Bivariate associations were assessed using Spearman’s correlation coefficients. Anhedonia and neurovegetative symptoms were considered the primary symptom-domain hypotheses based on prior literature, whereas analyses of other MADRS items were exploratory.

The following statistical tests were applied as appropriate: (a) independent-samples Student’s *t*-tests for comparisons of normally distributed continuous variables; (b) Mann–Whitney *U* tests for comparisons of non-normally distributed variables; (c) Fisher’s exact tests for categorical variables; and (d) multivariate linear regression (MLR) analyses to evaluate associations of inflammatory factors with depression severity and depressive symptom domains. Stepwise MLR analyses were performed. Clinically relevant covariates (age, sex, BMI, smoking status, medication use (antidepressant monotherapy vs. combined pharmacotherapy), and history of suicide attempt) were included as candidate predictors regardless of their bivariate associations, together with the depression variables of interest. Quantile regression (QR) was used to examine associations across the full distribution of circulating LPS levels and depressive symptoms, because we hypothesized that the effects would differ across the LPS distribution. Unlike ordinary least squares regression, QR does not assume normally distributed residuals and is robust to skewness and outliers, making it particularly suitable for biologically skewed variables such as LPS. Variance inflation factors (VIFs) were used to assess multicollinearity. QR models were estimated at the 5th, 10th, 25th, 50th, 75th, 90th, and 95th quantiles. Parameter estimates, confidence intervals, and *p*-values were based on 1000 bootstrap samples. QR coefficients were interpreted analogously to MLR coefficients but represent changes in specific quantiles of the dependent variable (LPS).

Mediation analyses were conducted to estimate indirect effects and determine whether associations between depression severity and circulating LPS were mediated by specific depressive symptoms. Indirect effects were estimated using a regression-based mediation framework. The total effect (c path), direct effect (c′ path), and indirect effect (a × b path) were calculated. The model was estimated in JASP, and indirect effects were tested using 1000 bootstrap samples to obtain 95% confidence intervals.

For all analyses, two-sided *p* < 0.05 was considered statistically significant. No correction for multiple testing was applied in order to minimize the risk of Type II error. Thus, results should be interpreted as hypothesis-generating.

## 3. Results

### 3.1. Sample Characteristics

The final study sample comprised 95 patients with MDD. The mean age was 43.8 years (SD = 14.07); 79.5% of participants were female; 38.9% were current smokers; and the mean BMI was 25.60 kg/m^2^ (SD = 5.01). The mean total MADRS score was 29.99 (95% CI: 28.41–31.57). All participants were receiving pharmacological treatment and were categorized into either antidepressant monotherapy (SSRI/SNRI/NaSSA) or combined pharmacotherapy (antidepressant/low-dose antipsychotic/mood stabilizer/anxiolytic) groups. The median duration of MDD was 8 years (Q1–Q3: 3–13.5), and the median duration of the current depressive episode was 6 months (Q1–Q3: 2.5–12).

### 3.2. Depression Severity and Symptoms’ Profile

Based on the MADRS cut-offs described above, 68 patients (71.6%) were classified as having mild-to-moderate depression (median 28 [Q1–Q3: 24–31]), while 27 patients (28.4%) were classified as having severe depression (median 38 [Q1–Q3: 37–41]). No significant differences were observed between the mild-to-moderate and severe MDD groups with respect to age, sex, BMI, duration of MDD, duration of the current depressive episode, or medication use (antidepressant monotherapy vs. combined pharmacotherapy) (all *p* > 0.05). Analysis of MADRS items showed that patients with severe depression demonstrated significantly higher levels of anhedonia, as measured by both MADRS item 8 (‘inability to feel’) and the anhedonia factor score (sum of MADRS items 1, 2, 6, 7 and 8), and severely depressed patients also had significantly disrupted appetite and sleep (MADRS items 4 and 5) (all *p*’s < 0.001; Table 1). In contrast, circulating LPS concentrations were significantly lower in the severe depression group than in the mild-to-moderate group (median difference = 52.35 (95% CI: 13.2–104.73), *p* = 0.007), whereas cytokine levels (IL-6, TNF-α, IFN-γ, and IL-10) did not differ significantly between severity groups (all *p*’s > 0.05).

### 3.3. Symptom-Specific Associations with Circulating LPS

Correlation analyses with clinical variables showed that LPS levels were significantly negatively correlated with severe MDD (ρ = −0.280, *p* < 0.05), MADRS item 8 (ρ = −0.286, *p* = 0.005) and anhedonia factor score (ρ = −0.349, *p* < 0.001) in MDD patients. This indicates that MDD patients with high LPS levels have a less severe form of depression and less anhedonia than MDD patients with low LPS levels. LPS levels were also negatively correlated with combined pharmacotherapy, which is consistent with these patients having less severe depression. Interestingly, LPS levels were not significantly correlated with IL-6, IL-10, IFN-γ or TNF-α levels, suggesting that LPS is an independent inflammatory marker in MDD patients (see Appendix A Table A1).

In MLR analyses (Table 2), circulating LPS levels were consistently and inversely associated with anhedonia. In the stepwise regression model, which included all MADRS items, age, sex, BMI, smoking status, medication use, and history of suicide attempt as candidate predictors, lower circulating LPS levels were independently predicted by the “inability to feel” item (MADRS item 8; β = −0.306, *p* = 0.001), reduced appetite (MADRS item 5; β = −0.252, *p* = 0.007), and combined pharmacotherapy (β = −0.323, *p* < 0.001).

### 3.4. Quantile Regression Analyses of Depressive Symptoms and LPS

Quantile regression analyses examining associations between individual MADRS symptoms and circulating LPS levels are presented in Table 3. Table 3 includes the results of seven multivariable regression models. The coefficients from QR should be interpreted in the context of 5th, 10th, 25th, 50th, 75th, 90th, and 95th percentiles. These multivariable analyses on circulating LPS demonstrated that symptom–LPS relationships varied substantially across the LPS distribution, with both the magnitude and direction of associations differing across quantiles.

Overall, the strongest and most consistent associations were observed at upper LPS quantiles (q = 0.75–0.95), whereas fewer and less stable effects were evident at median and lower quantiles. Model fit, assessed using pseudo-R^2^ values, also increased across higher quantiles, ranging from 0.302 to 0.398, indicating greater explanatory power among individuals with elevated LPS levels.

Among individual symptoms, MADRS item 5 (“reduced appetite”) and MADRS item 8 (“inability to feel”) demonstrated the most robust associations across multiple quantiles. Consistent with MLR findings, MADRS item 8 showed a stable inverse association with circulating LPS from the median through the highest quantiles and exhibited the largest effect size (B = −53.90, *p* < 0.001 at q = 0.95).

QR analyses also suggested symptom-specific variation in the associations between circulating LPS and individual MADRS items that was not apparent in mean-based models. At higher LPS quantiles, MADRS item 1 (“apparent sadness”), item 4 (“reduced sleep”), and item 6 (“concentration difficulties”) were positively associated with LPS. In contrast, MADRS item 3 (“inner tension”), item 5 (“reduced appetite”), item 7 (“lassitude”), item 8 (“inability to feel”) and item 10 (“suicidality”) were negatively associated with LPS.

### 3.5. Mediation Analysis: Role of Anhedonia and Neurovegetative Symptoms

Mediation analyses examined whether anhedonia and neurovegetative symptoms explained the relationship between higher depression severity and lower circulating LPS levels.

**Figure 1:**
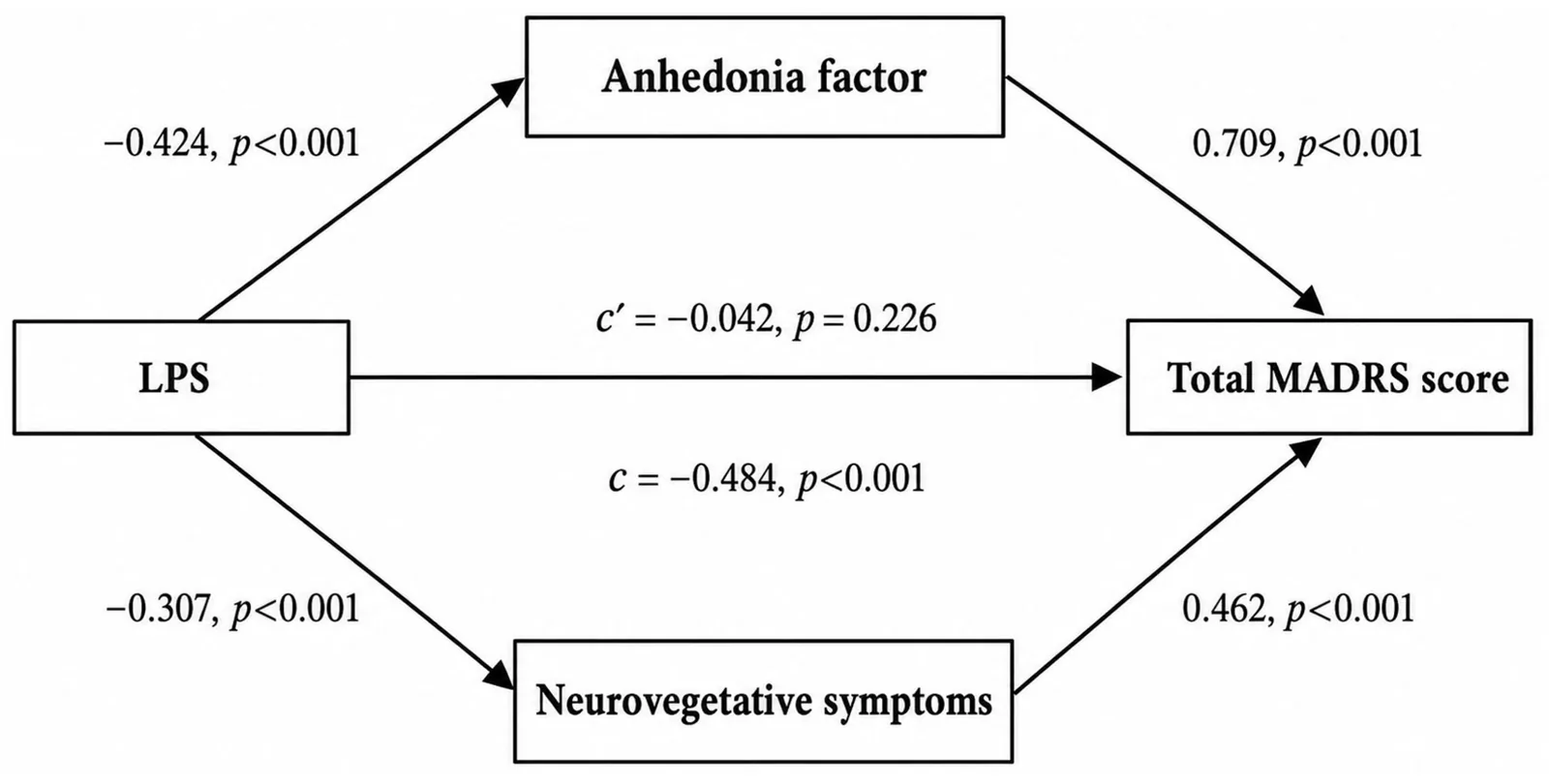
**Composite mediation model examining the association between depression severity and circulating LPS mediated by anhedonia factor and neurovegetative symptoms.** Coefficients shown are standardized. Abbreviations: MADRS—Montgomery–Åsberg Depression Rating Scale; LPS—lipopolysaccharide; anhedonia factor—sum of MADRS items 1 (‘apparent sadness’), item 2 (‘reported sadness’), item 6 (‘concentration difficulties’), item 7 (‘lassitude’), item 8 (‘inability to feel’); Neurovegetative symptoms—sum of MADRS items 4 + 5 (MADRS item 4—reduced sleep and MADRS item 5—reduced appetite).

In the composite mediation model (Figure 1), circulating LPS was significantly associated with lower anhedonia factor scores and fewer neurovegetative symptoms (MADRS items 4 + 5), while both higher anhedonia factor scores and greater neurovegetative symptoms were significantly associated with higher total MADRS scores (all *p* < 0.001). The total effect of LPS on depression severity was significant (β = −0.484, SE = 0.095, *p* < 0.001, 95% CI [−0.670, −0.298]). However, after inclusion of the mediators, the direct effect of LPS on depression severity was no longer significant (β = −0.042, SE = 0.035, *p* = 0.226, 95% CI [−0.110, 0.026]), indicating that the association was largely explained by indirect pathways. The indirect effect through the anhedonia factor was significant (β = −0.333, SE = 0.071, *p* < 0.001, 95% CI [−0.485, −0.124]), as was the indirect effect through neurovegetative symptoms (β = −0.142, SE = 0.046, *p* = 0.002, 95% CI [−0.230, −0.052]). The total indirect effect remained significant (β = −0.442, SE = 0.091, *p* < 0.001, 95% CI [−0.645, −0.246]), consistent with full mediation.

**Figure 2:**
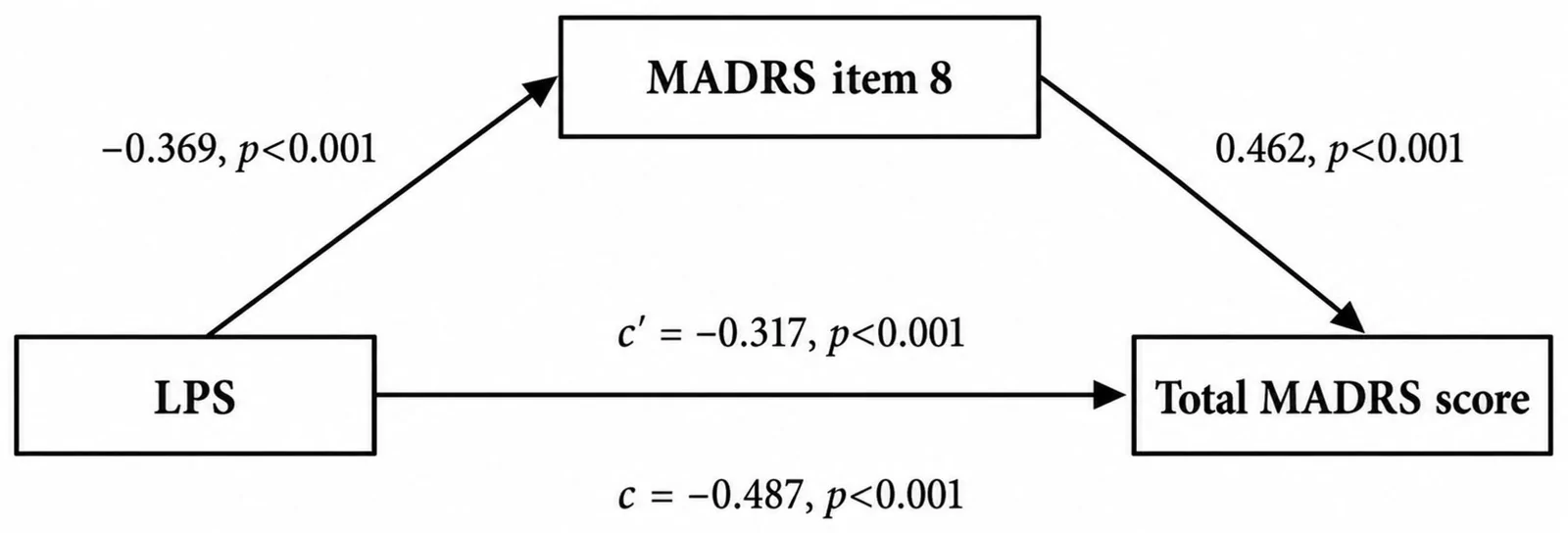
**Single-item mediation model examining the association between depression severity and circulating LPS mediated by MADRS item 8.** Coefficients shown are standardized. Abbreviations: MADRS—Montgomery–Åsberg Depression Rating Scale; LPS—lipopolysaccharide; MADRS item 8—‘inability to feel’.

In the single-item mediation model (Figure 2), circulating LPS was significantly associated with lower scores on MADRS item 8 (“inability to feel”), while higher item 8 scores were associated with greater depression severity (all *p* < 0.001). The total effect of LPS on depression severity was significant (β = −0.487, SE = 0.092, *p* < 0.001, 95% CI [−0.668, −0.307]). After inclusion of MADRS item 8 as a mediator, the direct effect remained significant (β = −0.317, SE = 0.085, *p* < 0.001, 95% CI [−0.484, −0.149]). The indirect effect through MADRS item 8 was significant (β = −0.171, SE = 0.055, *p* = 0.002, 95% CI [−0.279, −0.062]), indicating partial mediation. These findings suggest that the association between higher circulating LPS and lower depression severity is partly explained by lower levels of inability to feel. Adjustment for all MADRS items, age, sex and combined pharmacotherapy use did not alter the significance of the pathways or the presence of mediation.

To address the potential overlap between the mediator and outcome, we repeated the analysis using the MADRS total score, excluding item 8 as the outcome. The findings remained similar: the total effect of LPS remained significant (β = −0.470, SE = 0.093, *p* < 0.001, 95% CI [−0.652, −0.288]), and the indirect effect through item 8 also remained significant (β = −0.129, SE = 0.048, *p* = 0.007, 95% CI [−0.222, −0.035]). This suggests that the mediating role of item 8 is not solely due to its inclusion in the total MADRS score.

## 4. Discussion

To our knowledge, this is the first study to examine the relationship between circulating LPS levels and depressive symptoms in patients with MDD. Several key findings emerged. First, participants with more severe depression exhibited lower circulating LPS levels compared to participants with less severe depression, despite no significant differences in cytokine concentrations. Second, anhedonia was robustly associated with lower LPS levels across all analytical approaches, including MLR, QR and mediation. Mediation analyses demonstrated that anhedonia explained the association between severe depression and lower LPS, suggesting that this relationship is symptom-specific rather than driven by overall severity. Third, although multiple depressive symptoms showed associations with LPS, these relationships varied across the LPS distribution, and LPS associations were most pronounced at higher LPS concentrations. This is consistent with LPS being elevated only in about a quarter of MDD patients, and these high-LPS patients having a distinct symptom profile.

If high LPS is associated with a distinct symptom profile in MDD, it is worth comparing whether injection of LPS into healthy human volunteers induces a similar symptom profile. It has been reported that 0.4–0.8 ng/kg LPS induces the following depressive symptoms when injected into healthy human volunteers: depressed mood [24,43,44], anxiety [43], reduced food intake [45,46], disrupted sleep [47], fatigue [48] and impaired memory [43]. In contrast, DellaGiola & Hannestad [49] reported that LPS did not induce anhedonia or feelings of worthlessness, hopelessness, guilt, or suicidal ideation. Note, however, that there are individual differences in LPS-induced mood changes [50]. Thus, there is similarity (but some differences) in the depressive symptoms induced acutely by injection of LPS (listed above) and those seen in MDD patients with high circulating LPS (i.e., more apparent sadness, concentration difficulties, and reduced sleep, but less inability to feel, inner tension, reduced appetite, lassitude, and suicidality).

However, the effects of acute LPS exposure may differ substantially from those of chronic low-grade LPS exposure. Acute LPS exposure induces extensive transcriptomic, epigenetic, metabolic, and functional reprogramming of innate immune cells, leading to long-lasting alterations in the response to subsequent LPS exposure. Depending on the dose, timing, and experimental context, these adaptations may result in either a diminished inflammatory response (endotoxin tolerance) or an enhanced response (endotoxin sensitization and/or trained immunity) [51,52,53,54,55]. Endotoxin tolerance can reduce LPS levels by upregulating LPS removal from the blood by multiple mechanisms [53,54,55,56]. This raises the possibility that the relation between LPS and MDD severity might be explained in terms of disease progression, i.e., MDD is first induced by LPS exposure, but this initial, milder form of MDD progresses over time to severe MDD in which LPS is downregulated. However, a simpler explanation is that MDD comprises biologically distinct subtypes, one of which is characterized by elevated LPS levels and a less severe clinical presentation, including lower levels of anhedonia. Severe MDD is known to have a range of qualitative differences from milder forms of MDD [4,57,58,59,60], and prior evidence indicates that only a subset of patients with MDD exhibit inflammatory dysregulation [1,3,61,62].

An alternative explanation for the observed lower circulating LPS levels in severe depression is reduced food intake. In our regression analyses, reduced appetite was independently associated with lower circulating LPS levels. Because gut-derived LPS is influenced by dietary intake, dietary patterns, and gut microbial dynamics [63,64,65,66], reduced food intake may decrease circulating LPS concentrations independently of a distinct biological subtype of MDD. However, the cross-sectional design precludes conclusions regarding causality or directionality.

The QR analyses were consistent with the presence of a subpopulation of high LPS patients with a different symptom profile. If the MDD population were homogeneous, but with different LPS levels associated with specific symptoms, we would expect the same symptom–LPS associations to be observed across the full range of LPS levels. However, we found consistent symptom associations only among a subpopulation of MDD patients with high LPS levels. Alternatively, there may be a threshold level of LPS required to induce specific depressive symptoms.

In clinical MDD populations, antidepressants, antipsychotics, and mood stabilizers have shown modest and variable effects on inflammatory biomarkers, often without consistent symptom-level improvements [67,68,69,70,71]. In the present study, medication use did not differ between depression severity groups. Although combined pharmacotherapy use was associated with lower circulating LPS in MLR, it showed no effect in mediation analyses, indicating that it did not influence the relationship between anhedonia and LPS. This pattern is consistent with experimental endotoxemia findings, where pharmacological interventions affect symptom expression and inflammatory biomarkers in a non-uniform manner [20,72,73]. Notably, no participants in the present study received treatments with established antianhedonic effects (e.g., vortioxetine, ketamine, agomelatine, or transcranial magnetic stimulation) [38,39,74,75,76], which may limit interpretation regarding treatment-specific effects.

In contrast to LPS, circulating TNF-α, IL-6, IL-10, and IFN-γ were not associated with individual depressive symptoms and depression severity in the present study. Previous studies investigating associations between inflammatory cytokines and specific depressive symptoms have yielded inconsistent results. Although elevated concentrations of pro-inflammatory cytokines have been associated with neurovegetative symptom dimensions, including sleep disturbance and appetite changes, in some studies [28,77,78], associations with core affective symptoms have been less consistent [79,80,81,82]. These discrepancies may reflect the marked heterogeneity of MDD, differences in depression severity, antidepressant treatment status, comorbidities, lifestyle factors, and methodological differences in symptom assessment and cytokine measurement, including the use of serum versus plasma and assay variability [1,83].

This study should be interpreted as exploratory due to its modest sample size, cross-sectional design, and multiple symptom-level analyses. The QR analyses involved multiple statistical comparisons (70 tests in total). At a significance threshold of α = 0.05, approximately 3–4 statistically significant findings would be expected by chance alone under the global null hypothesis. As this study was exploratory and hypothesis-generating, we did not apply formal correction for multiple comparisons to avoid increasing the risk of Type II error. Given the sample size, estimates at the extreme quantiles are based on relatively few observations and are therefore less stable and subject to greater uncertainty. Therefore, isolated findings, particularly those observed at a single quantile, should be interpreted cautiously and require confirmation in independent studies.

The cross-sectional design of the present study limits the ability to establish temporal or causal relationships between inflammatory biomarkers and depressive symptoms. A longitudinal study would be more useful for assessing the causal role of LPS and its potential relationship with disease progression. Additionally, studies examining the causes of elevated LPS would be useful for identifying potential treatment targets. LPS was measured by ELISA, which, although sensitive, does not detect all forms of LPS, and only some forms of LPS are inflammatory [12]. Residual confounding from factors not fully assessed in the present study, including dietary habits and metabolic status, cannot be excluded, as these factors may influence circulating inflammatory biomarker levels [63,64,65,66,84,85]. Finally, depressive symptoms were assessed using the MADRS, which, although widely used, may not fully capture atypical or somatic symptoms.

These findings have several important translational implications. First, they support the concept of biologically heterogeneous depression, where LPS is relevant only in a subset of MDD patients. Second, they suggest that anhedonia may serve as a clinically accessible marker of underlying biological differences, potentially guiding stratification in future studies. Third, the findings justify testing whether lowering LPS levels in this MDD population can reduce depressive symptoms. Potential ways to reduce LPS levels include treating gut dysbiosis, treating ‘leaky gut’, ingesting LPS-degrading enzymes, or vaccination with non-toxic forms of LPS [55,83,86,87,88].

## 5. Conclusions

This study suggests that circulating LPS levels are higher in a subset of patients with MDD characterized by a distinct symptom and severity profile, including less pronounced anhedonia. Associations between LPS levels and depressive symptoms were most evident among patients with higher LPS levels, supporting the presence of biological heterogeneity within MDD.

## Figures and Tables

**Table 1:** Depressive symptom measures and inflammatory factors grouped by depression severity in MDD patients.

	Mild-to-Moderate Depression n = 68	Severe Depression n = 27	Median Difference ^a^	*p*
Depressive symptom measures Median (Q1–Q3)				
Anhedonia factor	17 (15.75–19)	22 (19.5–23.5)	−5 (−9; −3)	<0.001
Anhedonia MADRS item 8	3 (2–4)	4 (3–4)	−1 (−1; 0)	<0.001
Neurovegetative symptoms	3 (2–4)	8 (5.5–9.5)	−4 (−6; −3)	<0.001
Inflammatory factors, pg/mL, Median (Q1–Q3)				
LPS	165.23 (90.90–278.90)	95.58 (61.90–155.80)	52.35 (13.2–104.73)	0.007
IL-6	1.24 (0.63–2.49)	1.51 (0.52–3.63)	−0.04 (−0.83–0.52)	0.837
TNF-α	1.92 (1.59–2.23)	1.92 (1.43–2.18)	0.10 (−0.19–0.36)	0.552
IFN-γ	7.91 (4.98–11.15)	8.27 (6.91–13.64)	−1.09 (−3.40–1.09)	0.330
IL-10	1.90 (0.93–3.84)	2.28 (1.38–3.52)	−0.16 (−1.04–0.62)	0.656

^a^ Mann–Whitney U test; Independent-Samples Hodges–Lehmann Median Difference (95% CI). Abbreviations: Q1—first quartile; Q3—third quartile; anhedonia factor—sum of MADRS items 1 (apparent sadness), item 2 (reported sadness), item 6 (concentration difficulties), item 7 (lassitude), item 8 (inability to feel); neurovegetative symptoms—sum of MADRS items 4 + 5 (MADRS item 4—reduced sleep and MADRS item 5—reduced appetite); MADRS item 8—inability to feel; LPS—lipopolysaccharide, IL-6—interleukin-6, TNF-α—tumour necrosis factor alpha, IFN-γ—interferon-gamma, IL-10—interleukin-10, MADRS—Montgomery–Åsberg Depression Rating Scale.

**Table 2:** Multiple linear regression models examining associations between depressive symptoms and circulating LPS levels.

Model	Predictor	Unstandardized Coefficient B	SE	Standardized Coefficient β	t	*p*	VIF
Model 1	MADRS item 8	−34.994	10.372	−0.306	−3.374	0.001	1.04
	Pharmacotherapy use (0; 1)	−93.521	26.223	−0.323	−3.566	<0.001	1.03
	MADRS item 5	−15.557	5.639	−0.252	−2.759	0.007	1.05
Model 2	Anhedonia factor	−10.495	2.683	−0.360	−3.912	<0.001	1.01
	Pharmacotherapy use (0; 1)	−80.445	26.710	−0.278	−3.012	0.003	1.01
Model 3	MADRS item 8	−39.895	10.580	−0.349	−3.771	<0.001	1.01
	Pharmacotherapy use (0; 1)	−82.189	26.814	−0.284	−3.065	0.003	1.01

Dependent variable: circulating lipopolysaccharide concentration. Models were estimated using a stepwise regression procedure, with age, sex, body mass index, smoking status, pharmacotherapy use (0 = antidepressant monotherapy, 1 = combined pharmacotherapy), and history of suicide attempt included as candidate predictors. Model 1 included all MADRS items as candidate predictors (model fit: R^2^ = 0.253, F = 11.63, *p* < 0.001); Model 2 included the anhedonia factor as the candidate predictor (model fit: R^2^ = 0.208, F = 13.23, *p* < 0.001); Model 3 included MADRS item 8 as the candidate predictor (model fit: R^2^ = 0.217, F = 12.73, *p* < 0.001). Abbreviations: MADRS—Montgomery–Åsberg Depression Rating Scale; anhedonia factor—sum of MADRS items 1 (‘apparent sadness’), item 2 (‘reported sadness’), item 6 (‘concentration difficulties’), item 7 (‘lassitude’), item 8 (‘inability to feel’); MADRS item 5—reduced appetite; MADRS item 8—inability to feel; SE—standard error; VIF—variance inflation factor.

**Table 3:** Quantile regression coefficients for associations between depressive symptoms and circulating LPS levels across the LPS distribution.

Unstandardized Coefficient B (95% CI)								
	Quantile	q = 0.05	q = 0.1	q = 0.25	q = 0.5	q = 0.75	q = 0.9	q = 0.95
MADRS Item								
1. Apparent sadness		1.2 (−28.0–30.4)	8.6 (−6.3–23.5)	12.9 (−12.6–38.3)	9.3 (−35.9–54.4)	1.2 (−49.5–52.0)	45.8 (−6.2–98.0)	45.7 * (1.6–89.8)
2. Reported sadness		−9.8 (−31.7–12.1)	3.5 (−7.7–14.7)	−10.8 (−29.9–8.4)	0.2 (−33.8–34.2)	3.0 (−35.3–41.2)	−20.7 (−60.0–18.5)	−1.0 (−34.3–32.2)
3. Inner tension		4.2 (−12.9–21.3)	−12.6 ** (−21.3–−3.9)	−1.2 (−16.1–13.7)	1.5 (−25.0–27.9)	−0.2 (−30.0–29.5)	−36.2 * (−66.7–−5.7)	−41.6 * (−67.5–−15.7)
4. Reduced sleep		−3.8 (−16.9–9.3)	−0.1 (−6.7–6.6.)	−2.0 (−13.4–9.4)	−17.0 (−37.2–3.3)	−8.3 (−31.1–14.5)	2.3 (−21.1–25.7)	21.1* (1.3–41.0)
5. Reduced appetite		−8.7 (−19.7–2.2)	−9.1 ** (−14.6–−3.6)	−6.5 (−16.0–3.0)	−5.6 (−22.5–11.3)	−14.7 (−33.7–4.3)	−25.7 * (−45.1–−6.2)	−32.5 *** (−49.1–−16.0)
6. Concentration difficulties		−4.3 (−22.9–14.3)	−5.8 (−15.2–3.7)	−13.4 (−29.6–2.8)	−8.9 (−37.7–19.8)	10.5 (−21.8–42.8)	26.7 (−6.5–59.8)	36.3 * (8.1–64.4)
7. Lassitude		3.6 (−15.5–22.6)	−6.4 (−16.1–3.3)	−6.7 (−23.3–9.9)	−17.2 (−46.7–12.3)	−29.4 (−62.5–3.8)	−27.3 (−61.3–6.7)	−40.5 ** (−69.4–−11.7)
8. Inability to feel		−11.6 (−33.4–10.2)	−21.4 *** (−32.5–−10.3)	−7.4 (−26.4–11.7)	−37.3 * (−71.1–−3.5)	−37.5 (−75.5–0.4)	−44.3 * (−83.3–−5.5)	−53.9 ** (−86.9–−20.9)
9. Pessimistic thoughts		−9.1 (−25.2–6.9)	−8.3 * (−16.5–−0.2)	−12.5 (−26.5–1.5)	−1.4 (−26.3–23.4)	−5.5 (−33.4–22.5)	12.5 (−16.1–41.2)	0.6 (−23.7–24.9)
10. Suicidality		8.2 (−25.2–6.9)	11.2 ** (4.1–18.3)	9.8 (−2.4–21.9)	−5.5 (−27.1–16.1)	−13.3 (−37.6–11.0)	−33.1 ** (−58.0–−8.2)	−21.8 * (−42.9–−0.7)
pseudo-R^2^		0.099	0.122	0.122	0.138	0.224	0.302	0.398

Values represent unstandardized regression coefficients (B). Models were adjusted for all other MADRS items. Positive coefficients indicate higher LPS levels with increasing symptom severity; negative coefficients indicate lower LPS levels. Abbreviations: q—quantile; MADRS—Montgomery–Åsberg Depression Rating Scale; 95% CI—95% Confidence Interval. * *p* < 0.05; ** *p* <0.01, *** *p* < 0.001.

## Data Availability

The datasets used and/or analysed during the current study are available from the corresponding author on reasonable request due to privacy and ethical restrictions.

## Abbreviations

The following abbreviations are used in this manuscript:

MDD	Major depressive disorder
MADRS	Montgomery–Åsberg Depression Rating Scale
LPS	Lipopolysaccharide
IL-6	Interleukin 6
TNF-α	Tumor necrosis factor alpha
IFN-γ	Interferon gamma
IL-10	Interleukin 10
QR	Quantile regression

## Appendix A

**Table A1:** Spearman’s correlation coefficients for predictors and outcome (LPS) variables in MDD patients.

	Variables	1	2	3	4	5	6	7	8	9	10	11	12	13
1	LPS	1												
2	IL-6	0.02	1											
3	TNF-α	0.11	−0.16	1										
4	IFN-γ	−0.15	0.13	0.16	1									
5	IL-10	−0.04	0.15	−0.21 *	0.01	1								
6	Pharmacotherapy	−0.26 *	0.07	−0.11	−0.11	0.03	1							
7	Age	−0.09	0.14	−0.06	−0.03	−0.10	0.16	1						
8	Gender	−0.01	−0.04	0.15	−0.05	−0.01	0.11	−0.03	1					
9	Duration	−0.05	−0.02	0.07	−0.11	−0.17	−0.11	0.30 *	−0.10	1				
10	Episode	0.04	−0.13	0.23 *	0.06	−0.02	−0.14	0.01	0.04	0.08	1			
11	Suicide	−0.02	−0.02	−0.04	0.06	0.13	0.04	−0.34 *	0.10	−0.09	−0.09	1		
12	Smoking	0.08	0.18	−0.09	0.09	−0.01	0.08	−0.24 *	−0.12	−0.14	−0.01	0.17	1	
13	BMI	0.01	0.26 *	−0.03	−0.11	−0.10	0.07	0.20 *	0.02	0.15	0.12	−0.07	−0.15	1
14	Severe MDD	−0.28 *	0.02	−0.06	0.10	0.05	0.01	0.07	0.18	−0.03	−0.16	0.25 *	−0.07	−0.06

* *p* < 0.05 (two tailed); LPS—lipopolysaccharide; IL-6—interleukin-6; TNF-α—tumor necrosis factor alpha; IFN-γ—interferon gamma; IL-10—interleukin-10; pharmacotherapy—medication use; duration—total major depressive disorder (MDD) duration, years; episode—duration of current depressive episode, months; suicide—history of suicide attempt, smoking—current smoking; BMI—body mass index; severe MDD—MDD with Montgomery–Åsberg Depression Rating Scale (MADRS) score 35–60.

## References

[1] Poletti S., Mazza M.G., Benedetti F. (2024). Inflammatory mediators in major depression and bipolar disorder. Transl. Psychiatry.

[2] Kouba B.R., de Araujo Borba L., Borges de Souza P., Gil-Mohapel J., Rodrigues A.L.S. (2024). Role of inflammatory mechanisms in major depressive disorder: From etiology to potential pharmacological targets. Cells.

[3] Yin Y., Ju T., Zeng D., Duan F., Zhu Y., Liu J., Li Y., Lu W. (2024). “Inflamed” depression: A review of the interactions between depression and inflammation and current anti-inflammatory strategies for depression. Pharmacol. Res..

[4] Cui L., Li S., Wang S., Wu X., Liu Y., Yu W., Wang Y., Tang Y., Xia M., Li B. (2024). Major depressive disorder: Hypothesis, mechanism, prevention and treatment. Signal Transduct. Target. Ther..

[5] Osimo E.F., Stochl J., Zammit S., Lewis G., Jones P.B., Khandaker G.M. (2020). Longitudinal population subgroups of CRP and risk of depression in the ALSPAC birth cohort. Compr. Psychiatry.

[6] Peacock B.N., Scheiderer D.J., Kellermann G.H. (2017). Biomolecular aspects of depression: A retrospective analysis. Compr. Psychiatry.

[7] Liu F.R., Yang L.Y., Zheng H.F., Zhou Y., Chen B.B., Xu H., Zhang Y.W., Shen D.Y. (2020). Plasma levels of Interleukin 18 but not amyloid-β or Tau are elevated in female depressive patients. Compr. Psychiatry.

[8] Park B.S., Lee J.O. (2013). Recognition of lipopolysaccharide pattern by TLR4 complexes. Exp. Mol. Med..

[9] Zamyatina A., Heine H. (2020). Lipopolysaccharide recognition in the crossroads of TLR4 and caspase-4/11 mediated inflammatory pathways. Front. Immunol..

[10] Na K., Oh B.C., Jung Y. (2023). Multifaceted role of CD14 in innate immunity and tissue homeostasis. Cytokine Growth Factor Rev..

[11] De Chiara S., De Simone Carone L., Cirella R., Andretta E., Silipo A., Molinaro A., Mercogliano M., Di Lorenzo F. (2025). Beyond the Toll-Like Receptor 4. Structure-dependent lipopolysaccharide recognition systems: How far are we?. ChemMedChem.

[12] Tume R., El Sherbiny S., Bono R., Gautier T., de Barros J.P.P., Meroño T. (2025). The balance between proinflammatory, “bad”, and immunomodulatory, “good”, lipopolysaccharide for understanding gut-derived systemic inflammation. Front. Immunol..

[13] Markov D.D., Zozulya S.A., Dolotov O.V. (2026). Inflammatory models of depression in rodents and humans. Front. Psychiatry.

[14] Yin R., Zhang K., Li Y., Tang Z., Zheng R., Ma Y., Chen Z., Lei N., Xiong L., Guo P. (2023). Lipopolysaccharide-induced depression-like model in mice: Meta-analysis and systematic evaluation. Front. Immunol..

[15] Pienaar L., Baijnath S., Millen A.M.E. (2024). Unravelling the neuroinflammatory links in depression: The potential of a lipopolysaccharide preclinical model. Discov. Med..

[16] Zhao X., Cao F., Liu Q., Li X., Xu G., Liu G., Zhang Y., Yang X., Yi S., Xu F. (2019). Behavioral, inflammatory and neurochemical disturbances in LPS and UCMS-induced mouse models of depression. Behav. Brain Res..

[17] Haba R., Shintani N., Onaka Y., Wang H., Takenaga R., Hayata A., Baba A., Hashimoto H. (2012). Lipopolysaccharide affects exploratory behaviors toward novel objects by impairing cognition and/or motivation in mice: Possible role of activation of the central amygdala. Behav. Brain Res..

[18] Lasselin J., Lekander M., Axelsson J., Karshikoff B. (2020). Fatigue and sleepiness responses to experimental inflammation and exploratory analysis of the effect of baseline inflammation in healthy humans. Brain Behav. Immun..

[19] Savitz J., Figueroa-Hall L.K., Kent Teague T., Drevets W.C. (2025). Systemic inflammation and anhedonic responses to an inflammatory challenge in adults with major depressive disorder: A randomized controlled trial. Am. J. Psychiatry.

[20] DellaGioia N., Devine L., Pittman B., Hannestad J. (2013). Bupropion pre-treatment of endotoxin-induced depressive symptoms. Brain Behav. Immun..

[21] Lasselin J., Benson S., Hebebrand J., Boy K., Weskamp V., Handke A., Hasenberg T., Remy M., Föcker M., Unteroberdörster M. (2020). Immunological and behavioral responses to in vivo lipopolysaccharide administration in young and healthy obese and normal-weight humans. Brain Behav. Immun..

[22] Benson S., Brinkhoff A., Lueg L., Roderigo T., Kribben A., Wilde B., Witzke O., Engler H., Schedlowski M., Elsenbruch S. (2017). Effects of acute systemic inflammation on the interplay between sad mood and affective cognition. Transl. Psychiatry.

[23] Benson S., Engler H., Wegner A., Rebernik L., Spreitzer I., Schedlowski M., Elsenbruch S. (2017). What makes you feel sick after inflammation? Predictors of acute and persisting physical sickness symptoms induced by experimental endotoxemia. Clin. Pharmacol. Ther..

[24] Eisenberger N.I., Inagaki T.K., Rameson L.T., Mashal N.M., Irwin M.R. (2009). An fMRI study of cytokine-induced depressed mood and social pain: The role of sex differences. Neuroimage.

[25] Stevens B.R., Goel R., Kim S., Richards E.M., Holbert R.C., Pepine C.J., Raizada M.K. (2018). Increased human intestinal barrier permeability plasma biomarkers zonulin and FABP2 correlated with plasma LPS and altered gut microbiome in anxiety or depression. Gut.

[26] Zhou Z., Guille C., Ogunrinde E., Liu R., Luo Z., Powell A., Jiang W. (2018). Increased systemic microbial translocation is associated with depression during early pregnancy. J. Psychiatr. Res..

[27] Wu H., Wang J., Teng T., Yin B., He Y., Jiang Y., Liu X., Yu Y., Li X., Zhou X. (2023). Biomarkers of intestinal permeability and blood-brain barrier permeability in adolescents with major depressive disorder. J. Affect. Disord..

[28] Křenek P., Hořínková J., Bartečků E. (2023). Peripheral inflammatory markers in subtypes and core features of depression: A systematized review. Psychopathology.

[29] Bayes A., Weickert T.W., Parker G., Spoelma M.J., North H.F., Lam-Po-Tang J., Weickert C.S. (2024). Peripheral inflammatory markers in melancholic versus non-melancholic depression. Psychoneuroendocrinology.

[30] Milasauskiene E., Burkauskas J., Jesmanas S., Gleizniene R., Borutaite V., Raskauskiene N. (2026). Lipopolysaccharide and Cytokine levels differ by disease severity in Major Depressive Disorder. Discov. Ment. Health.

[31] Milasauskiene E., Burkauskas J., Jesmanas S., Gleizniene R., Borutaite V., Skemiene K., Vaitkiene P., Adomaitiene V., Lukosevicius S., Gradauskiene B. (2024). The links between neuroinflammation, brain structure and depression: A cross-sectional study protocol. PLoS ONE.

[32] Williams J.B., Kobak K.A. (2008). Development and reliability of a structured interview guide for the Montgomery-Åsberg Depression Rating Scale (SIGMA). Br. J. Psychiatry.

[33] Bunevicius A., Staniute M., Brozaitiene J., Pommer A.M., Pop V.J.M., Montgomery S.A., Bunevicius R. (2012). Evaluation of depressive symptoms in patients with coronary artery disease using the Montgomery Åsberg Depression Rating Scale. Int. Clin. Psychopharmacol..

[34] Müller M.J., Szegedi A., Wetzel H., Benkert O. (2000). Moderate and severe depression: Gradations for the Montgomery-Åsberg Depression Rating Scale. J. Affect. Disord..

[35] Luca A., Luca M., Kasper S., Pecorino B., Zohar J., Souery D., Montgomery S., Ferentinos P., Rujescu D., Messina A. (2024). Anhedonia is associated with a specific depression profile and poor antidepressant response. Int. J. Neuropsychopharmacol..

[36] Nakonezny P.A., Morris D.W., Greer T.L., Byerly M.J., Carmody T.J., Grannemann B.D., Bernstein I.H., Trivedi M.H. (2015). Evaluation of anhedonia with the Snaith-Hamilton Pleasure Scale (SHAPS) in adult outpatients with major depressive disorder. J. Psychiatr. Res..

[37] Ameli R., Luckenbaugh D.A., Gould N.F., Holmes M.K., Lally N., Ballard E.D., Zarate C.A. (2014). SHAPS-C: The Snaith-Hamilton Pleasure Scale modified for clinician administration. PeerJ.

[38] Cao B., Park C., Subramaniapillai M., Lee Y., Iacobucci M., Mansur R.B., Zuckerman H., Phan L., McIntyre R.S. (2019). The efficacy of vortioxetine on anhedonia in patients with major depressive disorder. Front. Psychiatry.

[39] McIntyre R.S., Loft H., Christensen M.C. (2021). Efficacy of vortioxetine on anhedonia: Results from a pooled analysis of short-term studies in patients with major depressive disorder. Neuropsychiatr. Dis. Treat..

[40] Miller A.H. (2025). Advancing an inflammatory subtype of major depression. Am. J. Psychiatry.

[41] Engler H., Brinkhoff A., Wilde B., Kribben A., Rohn H., Witzke O., Schedlowski M., Benson S. (2023). Endotoxin-induced physiological and psychological sickness responses in healthy humans: Insights into the post-acute phase. Neuroimmunomodulation.

[42] Tukey J.W. (1977). Exploratory Data Analysis.

[43] Reichenberg A., Yirmiya R., Schuld A., Kraus T., Haack M., Morag A., Pollmächer T. (2001). Cytokine-associated emotional and cognitive disturbances in humans. Arch. Gen. Psychiatry.

[44] Irwin M.R., Cole S., Olmstead R., Breen E.C., Cho J.J., Moieni M., Eisenberger N.I. (2019). Moderators for depressed mood and systemic and transcriptional inflammatory responses: A randomized controlled trial of endotoxin. Neuropsychopharmacology.

[45] Reichenberg A., Kraus T., Haack M., Schuld A., Pollmächer T., Yirmiya R. (2002). Endotoxin-induced changes in food consumption in healthy volunteers are associated with TNF-alpha and IL-6 secretion. Psychoneuroendocrinology.

[46] van Eeden W.A., van Hemert A.M., Carlier I.V.E., Penninx B.W.J.H., Lamers F., Fried E.I., Schoevers R., Giltay E.J. (2020). Basal and LPS-stimulated inflammatory markers and the course of individual symptoms of depression. Transl. Psychiatry.

[47] Mullington J., Korth C., Hermann D.M., Orth A., Galanos C., Holsboer F., Pollmächer T. (2000). Dose-dependent effects of endotoxin on human sleep. Am. J. Physiol. Regul. Integr. Comp. Physiol..

[48] Kullmann J.S., Grigoleit J.S., Lichte P., Kobbe P., Rosenberger C., Banner C., Wolf O.T., Engler H., Oberbeck R., Elsenbruch S. (2013). Neural response to emotional stimuli during experimental human endotoxemia. Hum. Brain Mapp..

[49] DellaGioia N., Hannestad J. (2010). A critical review of human endotoxin administration as an experimental paradigm of depression. Neurosci. Biobehav. Rev..

[50] Cho J.H., Irwin M.R., Eisenberger N.I., Lamkin D.M., Cole S.W. (2019). Transcriptomic predictors of inflammation-induced depressed mood. Neuropsychopharmacology.

[51] Draisma A., Pickkers P., Bouw M.P.W.J.M., van der Hoeven J.G. (2009). Development of endotoxin tolerance in humans in vivo. Crit. Care Med..

[52] Barr A.M., Song C., Sawada K., Young C.E., Honer W.G., Phillips A.G. (2003). Tolerance to the anhedonic effects of lipopolysaccharide is associated with changes in syntaxin immunoreactivity in the nucleus accumbens. Int. J. Neuropsychopharmacol..

[53] López-Collazo E., del Fresno C. (2024). Endotoxin tolerance and trained immunity: Breaking down immunological memory barriers. Front. Immunol..

[54] Vergadi E., Vaporidi K., Tsatsanis C. (2018). Regulation of endotoxin tolerance and compensatory anti-inflammatory response syndrome by non-coding RNAs. Front. Immunol..

[55] Roszak K., Roy K., Sobocińska J., Spisz P., Jędrzejewski T., Wrotek S. (2025). Endotoxin’s impact on organism: From immune activation to tolerance and beyond. J. Clin. Med..

[56] Kumar P., Schroder E.A., Rajaram M.V.S., Harris E.N., Ganesan L.P. (2024). The battle of LPS clearance in host defense vs. inflammatory signaling. Cells.

[57] Zimmerman M., Balling C., Chelminski I., Dalrymple K. (2018). Understanding the severity of depression: Which symptoms of depression are the best indicators of depression severity?. Compr. Psychiatry.

[58] Berk M., Köhler-Forsberg O., Turner M., Penninx B.W.J.H., Wrobel A., Firth J., Loughman A., Reavley N.J., McGrath J.J., Momen N.C. (2023). Comorbidity between major depressive disorder and physical diseases: A comprehensive review of epidemiology, mechanisms and management. World Psychiatry.

[59] Chen S., Long X., Chen S., Zhang Y., Bi B., Dong Z. (2026). Comorbidity patterns and immune-metabolic differences in patients with acute depressive episodes. Compr. Psychiatry.

[60] Kuzior H., Fiebich B.L., Yousif N.M., Saliba S.W., Ziegler C., Nickel K., Maier S.J., Süß P., Runge K., Matysik M. (2020). Increased IL-8 concentrations in the cerebrospinal fluid of patients with unipolar depression. Compr. Psychiatry.

[61] Osimo E.F., Baxter L.J., Lewis G., Jones P.B., Khandaker G.M. (2019). Prevalence of low-grade inflammation in depression: A systematic review and meta-analysis of CRP levels. Psychol. Med..

[62] Kouba B.R., Choudhury A., Fiori L.M., Turecki G. (2024). Role of inflammatory mechanisms in major depressive disorder. Biomedicines.

[63] Fuke N., Nagata N., Suganuma H., Ota T. (2019). Regulation of gut microbiota and metabolic endotoxemia with dietary factors. Nutrients.

[64] Jawamis A., Al-Domi H., Al Sarayreh N. (2025). Effect of dietary fat intake on metabolic endotoxemia: Mechanisms and clinical insights. Clin. Nutr. ESPEN.

[65] Mohr A.E., Crawford M., Jasbi P., Fessler S., Sweazea K.L. (2022). Lipopolysaccharide and the gut microbiota: Considering structural variation. FEBS Lett..

[66] Mercogliano M., Mazziotti V., Silipo A., Molinaro A., Di Lorenzo F. (2026). The hidden language of gut-derived lipopolysaccharides: Fine chemistry, huge immunological consequences. Chem. Sci..

[67] Köhler C.A., Freitas T.H., Stubbs B., Maes M., Solmi M., Veronese N., de Andrade N.Q., Morris G., Fernandes B.S., Brunoni A.R. (2018). Peripheral alterations in cytokine and chemokine levels after antidepressant drug treatment for major depressive disorder: Systematic review and meta-analysis. Mol. Neurobiol..

[68] Strawbridge R., Arnone D., Danese A., Papadopoulos A., Herane Vives A., Cleare A.J. (2015). Inflammation and clinical response to treatment in depression: A meta-analysis. Eur. Neuropsychopharmacol..

[69] Nava R.G., Adri A.S., Filgueiras I.S., Nóbile A.L., Barcelos P.M., Corrêa Y.L.G., de Oliveira S.F., Cabral-Miranda G., Dias H.D., Schimke L.F. (2025). Modulation of neuroimmune cytokine networks by antidepressants: Implications in mood regulation. Transl. Psychiatry.

[70] Yin M., Zhou H., Li J., Wang L., Zhu M., Wang N., Yang P., Yang Z. (2025). The change of inflammatory cytokines after antidepressant treatment and correlation with depressive symptoms. J. Psychiatr. Res..

[71] Patel S., Keating B.A., Dale R.C. (2023). Anti-inflammatory properties of commonly used psychiatric drugs. Front. Neurosci..

[72] Hannestad J., DellaGioia N., Ortiz N., Pittman B., Bhagwagar Z. (2011). Citalopram reduces endotoxin-induced fatigue. Brain Behav. Immun..

[73] Rodrigues F.T.S., Souza M.R.M., Lima C.N.C., Silva F.E.R., Costa D.V.S., Santos C.C., Miyajima F., de Sousa F.C.F., Vasconcelos S.M.M., Barichello T. (2018). Major depression model induced by repeated and intermittent lipopolysaccharide administration: Long-lasting behavioral, neuroimmune and neuroprogressive alterations. J. Psychiatr. Res..

[74] Almeida T.M., Generoso I.P., Rosa D.A.A., Pinheiro T.B., Foletto L.D., Jorge G.M.T., Grilo L.B., da Silva U.R.L., Cordeiro Q., Uchida R.R. (2024). The anti-anhedonic effects of ketamine in the treatment of resistant unipolar and bipolar depression: A systematic review and meta-analysis of current data. J. Affect. Disord. Rep..

[75] Sonmez A.I., Webler R., Krueger A.M., Godoy-Henderson C., Sullivan C., Wilson S., Olsen S., Schmid S., Herman A., Widge A. (2024). Effects of TMS on anhedonia and suicidal ideation in treatment-resistant depression: Outcomes from the University of Minnesota Interventional Psychiatry Program. J. Mood Anxiety Disord..

[76] Guo P., Xu Y., Lv L., Feng M., Fang Y., Huang W.-Q., Cheng S.-F., Qian M.-C., Yang S., Wang S.-K. (2023). A multicenter, randomized controlled study on the efficacy of agomelatine in ameliorating anhedonia, reduced motivation, and circadian rhythm disruptions in patients with major depressive disorder (MDD). Ann. Gen. Psychiatry.

[77] Majd M., Saunders E.F.H., Engeland C.G. (2020). Inflammation and the dimensions of depression: A review. Front. Neuroendocrinol..

[78] Wijaya M.T., Jin R.R., Liu X., Zhang R., Lee T.M.C. (2022). Towards a multidimensional model of inflamed depression. Brain Behav. Immun. Health.

[79] Tang W., Liu H., Chen L., Zhao K., Zhang Y., Zheng K., Zhu C., Zheng T., Liu J., Wang D. (2021). Inflammatory cytokines, complement factor H and anhedonia in drug-naive major depressive disorder. Brain Behav. Immun..

[80] Lu S., Wu C., Jia L., Fang Z., Lu J., Mou T., Hu S., He H., Huang M., Xu Y. (2022). Increased plasma levels of IL-6 are associated with striatal structural atrophy in major depressive disorder patients with anhedonia. Front. Psychiatry.

[81] Zwiep J.C., Lamers F., Vinkers C.H., van der Wee N.J.A., Penninx B.W.J.H., Nawijn L., Milaneschi Y. (2026). Inflammation, metabolic dysregulation, and depression profiles related to anhedonia and atypical, energy-related symptoms. Brain Behav. Immun..

[82] Lamers F., Milaneschi Y., Penninx B.W.J.H., Dantzer R., Capuron L. (2018). Depression subtypes and inflammation: Atypical rather than melancholic depression is linked with immunometabolic dysregulations. Inflammation and Immunity in Depression: Basic Science and Clinical Applications.

[83] Gędek A., Modrzejewski S., Materna M., Iwański M., Wichniak A., Dominiak M. (2025). Altered cytokine levels in the first episode of major depression and in antidepressant-naïve patients: A systematic review and meta-analysis. Int. J. Mol. Sci..

[84] Ge Z., Hu Y., Kan W., Li L., Xu J., Zhang Y., Zheng N., Wang G., Du J. (2025). Lipid metabolic dysregulation-induced neuroinflammation in the pathophysiology of major depressive disorder. Front. Immunol..

[85] Meng X., Han L., Fu J., Hu C., Lu Y. (2025). Associations between metabolic syndrome and depression, and the mediating role of inflammation: Based on the NHANES database. J. Affect. Disord..

[86] Aleman R.S., Moncada M., Aryana K.J. (2023). Leaky gut and the ingredients that help treat it: A review. Molecules.

[87] Mohammad S., Thiemermann C. (2021). Role of metabolic endotoxemia in systemic inflammation and potential interventions. Front. Immunol..

[88] Bennett-Guerrero E., McIntosh T.J., Barclay G.R., Snyder D.S., Gibbs R.J., Mythen M.G., Poxton I.R. (2000). Preparation and preclinical evaluation of a novel liposomal complete-core lipopolysaccharide vaccine. Infect. Immun..

